# Macrophage Migration and Its Regulation by CSF-1

**DOI:** 10.1155/2012/501962

**Published:** 2012-02-15

**Authors:** Fiona J. Pixley

**Affiliations:** School of Medicine and Pharmacology, The University of Western Australia, M510, 35 Stirling Highway, Crawley, WA 6009, Australia

## Abstract

Macrophages are terminally differentiated cells of the mononuclear phagocytic lineage and develop under the stimulus of their primary growth and differentiation factor, CSF-1. Although they differentiate into heterogeneous populations, depending upon their tissue of residence, motility is an important aspect of their function. To facilitate their migration through tissues, macrophages express a unique range of adhesion and cytoskeletal proteins. Notably, macrophages do not form large, stable adhesions or actin stress fibers but rely on small, short lived point contacts, focal complexes and podosomes for traction. Thus, macrophages are built to respond rapidly to migratory stimuli. As well as triggering growth and differentiation, CSF-1 is also a chemokine that regulates macrophage migration via activation the CSF-1 receptor tyrosine kinase. CSF-1R autophosphorylation of several intracellular tyrosine residues leads to association and activation of many downstream signaling molecules. However, phosphorylation of just one residue, Y721, mediates association of PI3K with the receptor to activate the major motility signaling pathways in macrophages. Dissection of these pathways will identify drug targets for the inhibition of diseases in which macrophages contribute to adverse outcomes.

## 1. Introduction

Macrophages reside in almost every tissue of the body and, as a result of their adaptation to the different tissue microenvironments, adopt a diverse range of morphologies and carry out a variety of functions. Despite their heterogeneity, macrophages all originate from the pluripotent hematopoietic stem cell and, under the influence of hematopoietic growth factors, differentiate through several multipotent progenitor stages to lineage committed mononuclear phagocytic precursors in the bone marrow[1–3]. The mononuclear phagocyte system is comprised of the mononuclear phagocyte precursors, monoblasts, and promonocytes, as well as circulating monocytes and fully differentiated, tissue resident macrophages [1–4]. Colony-stimulating factor-1 (CSF-1) has long been recognized as the primary growth factor regulating the survival, proliferation, and differentiation of cells of the mononuclear phagocytic lineage [1, 3, 5]. It is also an essential differentiation factor for the bone resorbing osteoclast [6]. A spontaneously occurring inactivating mutation in the mouse CSF-1 gene (*osteopetrotic, Csf-1^op^*) is associated with reduced tissue macrophage numbers and a marked reduction in osteoclasts, and causes osteopetrosis along with other developmental defects [1, 7–9]. CSF-1 signals through the CSF-1 receptor tyrosine kinase (RTK), encoded by the c-fms proto-oncogene [10], to trigger a series of phosphorylation cascades that mediate cellular responses to CSF-1 [1]. While the phenotype of mice nullizygous for the CSF-1R (*Csf1r^−^/Csf1r^−^*) largely recapitulates that seen in the *Csf1^op^/Csf1^op^* mouse, it is more severe and the discrepancy has since been explained by the discovery of a second partially redundant ligand for the CSF-1R, interleukin-34 (IL-34) [11–13].

Macrophages are professionally motile cells that carry out a variety of roles in immune surveillance and normal tissue development by secreting cytokines and growth factors and phagocytosing foreign material and apoptotic cells. Transendothelial and interstitial motility is an essential aspect of their function as they must be able to move to specific sites upon demand. From studies in primary macrophages and CSF-1 dependent macrophage cell lines, it is evident that CSF-1 is not only a mononuclear phagocyte lineage growth factor but is an important regulator of macrophage motility [1, 14–16]. Depletion of specific subsets of tissue macrophages in the *Csf1^op^/Csf1^op^* mouse and their reconstitution upon restoration of CSF-1 expression indicates that CSF-1 regulates the differentiation and migration of trophic and/or scavenger macrophages that are physiologically important for normal development and tissue homeostasis rather than in immune function [3, 9, 11, 17]. CSF-1 or CSF-1R deficient mice demonstrate abnormal neural, skeletal, and glandular development, not only due to reduced macrophage and osteoclast numbers but also through reduced matrix remodeling [3]. Thus, CSF-1-induced motility is likely to be an important element of macrophage function in development. Beyond their critical physiological role, CSF-1 dependent macrophages have also been demonstrated to promote disease progression in conditions ranging from cancer to atherosclerosis and arthritis [1, 3, 18, 19]. Reactivation of developmental macrophage functions may underlie the progression of these pathologies [3]. To participate in the disease process, macrophages must first migrate to the affected tissue. Furthermore, in the case of enhancement of tumor invasion, tumor-associated macrophages and mammary carcinoma cells have been shown to migrate away from the primary tumor together [20]. Yet little is known about how macrophage motility is regulated, how the motility machinery differs from other cell types and whether inhibition of macrophage motility may improve disease outcomes. Moreover, CSF-1 activated signaling pathways activate molecules or protein isoforms selectively expressed in macrophages [1], some of which may be attractive therapeutic targets to specifically inhibit macrophage infiltration into sites of disease. Considering the contribution of macrophages and CSF-1 to tumour dissemination and the progression of several inflammatory disorders [3, 18, 19], this review focuses on our current understanding of macrophage migration and its regulation by CSF-1.

## 2. Macrophage Motility

Almost all cell types are capable of migration but, in the adult organism, motility is particularly important for cells participating in immune cell function and wound healing. Leukocytes move rapidly compared to other cells, with neutrophils and lymphocytes measured at speeds of up to 25–30 *μ*m/min [21, 22]. While macrophages are slower than other leukocytes, moving at ~1 *μ*m/min *in vitro*, *in vivo *they respond rapidly to wounding or inflammatory signals and can migrate over considerable distances. Indeed, their migration speed has been measured at over 10 *μ*m/min when attracted into a wound in a fish model [23]. Compared to fibroblasts and epithelial cells (~0.1–0.5 *μ*m/min) [21], macrophages are considered to be efficient migrators.

The fundamental locomotory mechanisms are broadly similar in most cell types [24, 25]. Motility is a complex and integrated process that has typically been broken down into five components: (1) cell polarization or breaking of symmetry upon designation of the leading edge, (2) actin polymerization-driven protrusion of the leading edge, (3) integrin-mediated adhesion of the extended protrusion to underlying extracellular matrix proteins to provide the necessary traction for (4) actomyosin contractility-based forward translocation of the cell body, and, finally, (5) de-adhesion of the trailing edge to complete the cycle [25, 26]. Nevertheless, this description is a simplification of an integrated process, for example actin polymerization and actomyosin contraction contribute to adhesion structure formation and maturation at the front of the cell and to their disassembly at the rear [27, 28] and adhesion strength affects protrusion and migration [29]. Furthermore, these processes most accurately describe a style of locomotion employed by mesenchymal cells such as fibroblasts and by endothelial cells. Leukocytes more commonly use a migration mode typified by the amoeba, *Dictyostelium discoides* [22]. The differences in amoeboid and mesenchymal migration are most clearly seen in 3D matrix environments where the interstitial matrix is preserved rather than digested and migrating cells do not appear to adhere to the matrix proteins in amoeboid migration [21, 22]. Indeed, recent work indicates that integrins are not required for interstitial migration of dendritic cells in the dermis or lymph nodes but are indispensable for transendothelial migration [30, 31]. Consistent with their intermediate migration speed, macrophages appear capable of both amoeboid and mesenchymal interstitial migration, depending on the structure and density of the surrounding matrix, as they can either propel themselves through loose connective tissue or actively digest a path through denser interstitial matrix [32, 33]. Moreover, matrix remodeling by tumor-associated macrophages promotes breast cancer cell invasion, indicating that macrophages normally digest extracellular matrix during interstitial migration [34].

**Figure 1:**
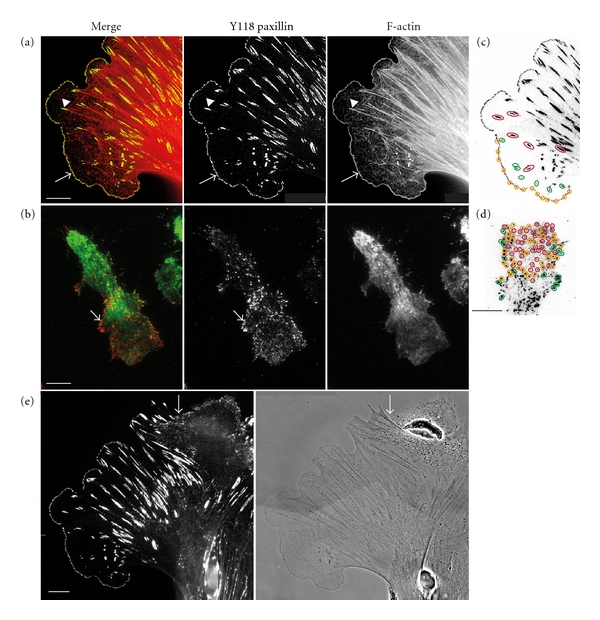
Macrophages are built for rapidly responsive migration. (a) A primary fibroblast, grown on a fibronectin coated coverslip in *α*+MEM, 10% FCS, and 120 ng/ml recombinant CSF-1, was fixed and stained for phosphoY118 paxillin (green) and F-actin (red). Phospho-Y118 paxillin staining eliminates background cytoplasmic staining of unphosphorylated paxillin [1, 16]. The arrow indicates focal complexes at the leading edge and the arrowhead indicates a focal adhesion giving rise to a stress fiber. (b) MacCsf1r^−/−^. WT macrophage, grown on a fibronectin coated coverslip in *α*+MEM, 10% FCS, and 120 ng/ml recombinant CSF-1, was fixed and stained for phosphoY118 paxillin (red) and F-actin (green) and examined by TIRF microscopy. The arrow indicates a focal complex giving rise to an F-actin cable. (c) Inverted image of phosphoY118 paxillin IF staining at the leading edge of the primary fibroblast, yellow circles indicate several nascent adhesions, green ovals highlight focal complexes and red ovals outline some focal adhesions. (d) Inverted image of phospho-Y118 paxillin IF staining in the leading lamellipodium of the macrophage, yellow circles indicate point contacts with strong phosphopaxillin staining, red circles indicate point contacts with moderate phosphopaxillin staining and green ovals outline the linear focal complexes. (e) A larger view of the fibroblast stained for pY118 paxillin (left) and shown by phase contrast (right) to demonstrate the co-cultured primary macrophage migrating underneath the fibroblast (arrow) and disrupting its focal adhesions. Note the lack of macrophage focal adhesions. Scale bars = 10 *μ*M.

Examination *in vitro* of the actin cytoskeleton and adhesion structures in macrophages and fibroblasts indicates important mechanistic differences in the motility machinery between the two cell types (Figure 1). As the leading edge of a fibroblast extends, it forms small nascent adhesions (Figures 1(a) and 1(c), yellow) that either quickly disappear or, if they are connected to actin microfilaments, cluster into small focal complexes just behind the leading edge (Figures 1(a), arrow; and 1(c), green). Then, as the fibroblast continues to move forward, the focal complexes in their turn either disappear or mature and coalesce into larger focal contacts (1–5 *μ*M) that anchor thick actin bundles or stress fibers (Figures 1(a), arrowhead; 1(c), red) [25, 35–37]. Indeed, the thickness of the bundled actin appears to control the size and shape of the underlying adhesion [36, 37]. In contrast to fibroblasts, macrophages form innumerable dot-like point contacts of varying phosphopaxillin content (Figures 1(b) and 1(d), yellow and red) under the ventral surface, most strikingly in the leading lamellipodium, along with scattered, mostly peripherally located focal complexes (Figure 1(d), green). Point contacts are also found in neuronal growth cones and highly motile cells [38] and resemble the widely distributed nascent adhesions of spreading fibroblasts after replating [39]. Macrophage adhesions do not mature into large focal contacts with attached stress fibers, although some focal complexes do anchor thin actin bundles (Figure 1(b), arrow) [16, 40]. Inverted phosphopaxillin immunofluorescent images clearly demonstrate the strikingly different pattern of adhesion at the leading edges of fibroblasts (Figure 1(c)) and macrophages (Figure 1(d)).

## 3. Macrophage Adhesions

Adhesions are multiprotein complexes that not only structurally link the cell adhesion receptors, integrins, to the actin cytoskeleton but also integrate and regulate a range of signals important for cell motility and growth [41]. The molecular associations and movement of individual components in the complexes are highly dynamic, allowing rapid responses to environmental and cellular cues [28, 42]. Tyrosine phosphorylation is an important regulatory mechanism for dynamic interplay of these components [43–45]. A number of tyrosine kinases localize to adhesions, including Src family kinases (SFK) and the adhesion kinases, focal adhesion kinase (FAK) and Pyk2, where they phosphorylate many adhesion proteins upon integrin engagement [41, 46]. Prominent among the phosphorylated adhesion proteins are the kinases themselves and paxillin, a highly phosphorylated multidomain scaffold protein that integrates and coordinates the regulation of adhesion signaling molecules, many of which control actin polymerization and actomyosin contractility [47]. Indeed, fluorescently tagged paxillin has been widely used to examine adhesion formation in living and fixed cells. Due to their small size and extensive ventral surface distribution, macrophage adhesion structures are difficult to visualize, as cytoplasmic paxillin almost completely obscures them. However, paxillin translocates to the plasma membrane and to adhesions when phosphorylated on Y31/Y118, so the use of phosphospecific paxillin antibodies and total internal reflection fluorescence microscopy (TIRF) greatly enhances our capacity to examine macrophage adhesions (Figure 1(b)) [16, 25, 48]. Despite their lack of large, longer lived adhesions, macrophages can be successfully cultured on glass and bacterial plastic. Moreover, the combined adhesive capacity of their collected point contacts and focal complexes enables macrophages to push underneath cocultured fibroblasts, disrupting the fibroblast's focal contacts in the process (Figure 1(e), arrow). Consistent with this observation, adhesion strength is positively correlated with the adhesive area of a cell and leading edge focal complexes have been shown to support a stronger traction force than mature focal contacts [35, 49]. Thus, it appears that individually weak adhesions in macrophages collectively give rise to robust but dynamic adhesion and, in combination with a readily remodeled actin cytoskeleton, permit rapidly responsive migration in macrophages.

**Figure 2:**
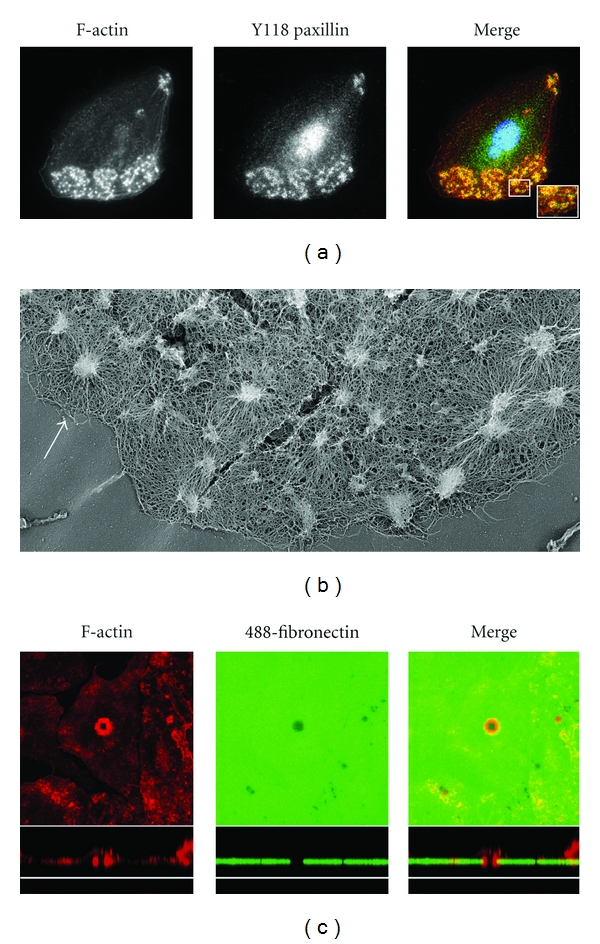
Macrophages form podosomes. (a) A human monocyte-derived macrophage, grown on a fibronectin coated coverslip in RPMI, 10% FCS, and 120 ng/ml of recombinant CSF-1, was fixed and stained for F-actin (red) and pY118 paxillin (green). Inset in the merge panel demonstrates the central F-actin-rich stud and the surrounding pY118 paxillin collar. (b) Cytoskeletal TEM preparation of a human monocyte-derived macrophage, grown on a glass coverslip, demonstrating many podosomes at the presumed leading edge. The arrow highlights a good example of a podosome containing a central dense actin column surrounded by radially orientated actin filament spokes. (c) Mouse bone marrow-derived macrophages were plated on Alexa-488 labeled fibronectin overlaying gelatin for 24 hours prior to fixation, staining for F-actin (red) and examination by confocal microscopy to demonstrate a podosomal rosette digesting the underlying matrix.

In addition to point contacts and focal complexes, macrophages also form podosomes, which are short-lived adhesion/motility organelles that consist of a dense core of actin surrounded by a collar of adhesion proteins (Figure 2(a)) [50, 51]. Cytoskeletal transmission EM studies indicate that podosomes have a distinctive hub and spoke microfilament architecture (Figure 2(b), arrow) [52]. In contrast to focal complexes, they are able to digest extracellular matrix and so are thought to be important for interstitial migration of macrophages and other myeloid cells [51, 53, 54]. It is not clear how podosomes contribute to motility but individual, short-lived podosomes often coalesce into higher order, more stable structures such as rosettes that efficiently digest the underlying matrix (Figure 2(c)) [55, 56]. Osteoclasts form large podosomal arrays or belts within which separate podosomes may become indistinct and which create the sealing zone necessary for effective bone resorption within the perimeter of this gasket [52]. Podosomes may also be important for leukocyte diapedesis, either between or through endothelial cells [57, 58]. Importantly, matrix digesting actin-rich rosette-like structures have been imaged in human primary macrophages within a 3D gelled collagen matrix, strongly suggesting podosomes play a role in macrophage migration *in vivo* [33].

Compared to actin-rich rosettes, imaging adhesion structures in living cells in 3D culture is difficult, leading to some doubt that they exist *in vivo*. However, by lowering the expression levels of genetically encoded fluorescently tagged adhesion proteins to reduce background cytoplasmic fluorescence, dynamic paxillin-rich 1 *μ*M cell-matrix adhesions were observed in the protrusions of U2OS osteosarcoma cells grown in 3D collagen gels [59]. These adhesions were demonstrated to form in contact with collagen fibers, suggesting adhesions are likely to be found *in vivo* in mesenchymally migrating cells. However, *in vivo *detection of adhesion structures will be extremely difficult, particularly in macrophages, which form such small adhesions in 2D culture systems.

## 4. CSF-1 Regulation of Macrophage Motility

Although CSF-1 was initially identified as a macrophage growth and differentiation factor [5], it was subsequently demonstrated to stimulate monocyte migration [14] and later studies confirmed that CSF-1 is a potent chemokinetic and chemotactic factor for macrophages [15]. Indeed, the pathophysiological importance of CSF-1-stimulated macrophage migration has recently been demonstrated in several diseases, including tumour invasion and metastasis [20, 60], inflammatory arthritis [61–63] and atherosclerosis [64, 65]. Tumor-associated macrophages secrete epidermal growth factor (EGF) and carcinoma cells secrete CSF-1 to set up a paracrine chemotactic loop that induces comigration of both cell types and promotion of invasion and metastasis [20, 59]. Inhibition of either EGF receptor or CSF-1R signaling prevents tumour cell motility *in vivo *[20]. Synovial macrophages have long been known to play a critical role in chronic rheumatoid arthritis and conventional therapies all reduce macrophage numbers in the synovium [66]. CSF-1, which is secreted by synoviocytes and endothelial cells, attracts monocytes to arthritic joints and stimulates their differentiation into inflammatory cytokine secreting macrophages and bone resorbing osteoclasts [66]. Importantly, selective CSF-1R inhibition significantly reduced joint infiltration and differentiation of macrophages in several autoimmune arthritis models with subsequent improvement in arthritis severity [63]. Downstream of the CSF-1R, a mutation causing reduced expression of PSTPIP2, a signaling protein that is selectively expressed in macrophages, results in an autoinflammatory disease [67, 68]. PSTPIP2 is tyrosine phoshorylated in response to CSF-1 and regulates ruffling, filopodia formation, and CSF-1-induced motility [67]. Thus CSF-1 regulation of macrophage migration is important in the development and progression of several diseases and elucidation of CSF-1-stimulated motility pathways is likely to identify possible therapeutic targets to modulate macrophage infiltrative capacity.

## 5. CSF-1R Signaling to Macrophage Motility

CSF-1 initially triggers membrane ruffling and spreading followed by increased formation of phosphotyrosine-rich adhesions and finally the macrophages polarize and begin to move [16, 40, 69]. CSF-1-stimulated actin polymerization is very rapid, with a sharp peak at 30 sec followed by a longer lasting wave at 3 min [69]. Polymerization is regulated by Rho family GTPases, Rac, Rho, and Cdc42, whose effectors include the Wiskott Aldrich syndrome protein (WASP)/WASP-family verprolin homologous (WAVE) family of actin nucleators [70, 71]. Increased focal complex and point contact formation is visible by 5 minutes but does not peak until 15 min after CSF-1 stimulation, coincident with maximal phosphorylation of paxillin by its adhesion kinases, Pyk2 and FAK [40]. Consistent with the importance of actin polymerization and adhesion formation in macrophage migration, macrophages deficient in Pyk2, FAK, WASP, or WAVE2 are poorly motile [48, 72–74]. However, the mechanisms by which CSF-1 stimulates actin polymerization and adhesion formation are not well understood and require careful dissection of the signaling pathways triggered by CSF-1R activation.

The effects of CSF-1 are mediated by the CSF-1R, a RTK of the platelet derived growth factor receptor (PDGFR) family. Upon binding of homodimeric CSF-1, the CSF-1R dimerizes, becomes activated and autophosphorylates at least 7 of its 20 intracellular tyrosine residues [1]. Phosphorylation of these tyrosine residues creates specific binding sites for phosphotyrosine (pTyr) binding domain-containing molecules and initiates a series of signaling cascades, leading to rapid stimulation of cytoskeletal remodeling and adhesion as well as gene transcription and protein translation [1, 75]. To identify pTyr CSF-1R-associated molecules and examine the specific pathways that mediate the various effects of CSF-1, earlier studies either ectopically expressed wild-type or tyrosine-to-phenylalanine (Y→F) mutant CSF-1Rs in fibroblasts or expressed chimeric receptors composed of a non-CSF-1R extracellular domain and Y→F mutated CSF-1R intracellular domains in myeloid cells. Results differed between fibroblast and myeloid cell studies, in part because mature macrophages selectively express specific proteins, isoforms or splice variants important for CSF-1R signaling [1, 67, 76]. To overcome these problems, we developed a system to express a single species of CSF-1R in a mature macrophage context. Immortalised macrophages derived from the CSF-1R^−/−^ mouse were transduced with either a wild-type or a tyrosine mutant CSF-1R [11, 77]. The Y-Eight-F (YEF) mutant CSF-1R, with eight tyrosine residues mutated to phenylalanine, is not phosphorylated in response to CSF-1 and macrophages expressing this receptor cannot survive in CSF-1 [77]. The system was used to examine loss-of-function effects of a panel of individual Y→F CSF-1R molecules and we have shown that phosphorylation of Y706 and Y721 in the kinase insert and Y974 at the C-terminus of the CSF-1R are important for normal macrophage morphology while juxta-membrane Y559 and activation loop Y807 are critical for macrophage proliferation and differentiation [77]. The YEF CSF-1R can be used as a backbone on which to add-back individual tyrosine residues. Add-back to the YEF CSF-1R of two known phosphotyrosine residues, Y559 and Y807, and a third, Y544, that may not be phosphorylated but is thought to be important for CSF-1R conformation, restores full proliferation in response to CSF-1 (unpublished results). Add-back of single tyrosine residues to the YEF receptor has been used to demonstrate that Y559 is the first residue phosphorylated in response to CSF-1 [78] and that it is necessary and sufficient for c-Cbl-mediated receptor ubiquitylation, full activation, and subsequent degradation of the receptor [78]. Individual tyrosine residues can also be added back to minimal proliferation competent add-back (AB) receptor, YEF. Y544, Y509, Y807 AB CSF-1R, to examine return-of-function signaling for the remaining pTyr residues.

Macrophages elongate when they polarize and begin to move [15, 79] so a loss of elongation can indicate reduced motility [40, 80]. Cells expressing the Y721F mutant receptor were apolar and previous studies in other cell lines had demonstrated a pY721-dependent association with the CSF-1R of two proteins known to signal to cell motility, phosphoinositide 3-kinase (PI3K) and phospholipase C (PLC)*γ*2 [81–83]. A detailed examination of the Y721F CSF-1R macrophages revealed a significant reduction in motility *in vitro* and, perhaps more importantly, these macrophages moved less well *in vivo* and there was a significant reduction in their capacity to enhance tumour cell invasion *in vitro* [69]. Underlying the reduction in motility was a loss of the first peak of CSF-1-stimulated actin polymerization and reduced paxillin phosphorylation and incorporation into adhesions. Add-back of Y721 to the YEF. Y544, Y559, Y807 AB CSF-1R restored actin polymerization and cell motility, indicating that pY721-based signaling regulates CSF-1-induced macrophage motility [69]. This system was also used to identify which of the two possible effectors, PI3K or PLC*γ*2, was responsible for initiating pY721-based motility signaling. While PLC*γ*2 associated with the activated CSF-1R in a pY721-independent manner, CSF-1 rapidly stimulated a prolonged Y721-dependent association of PI3K with the receptor, which resulted in PIP_3_ production [69]. Thus the primary mediator of CSF-1-stimulated motility in macrophages is PI3K.

Class IA PI3Ks consist of a p110 catalytic subunit bound to a p85 regulatory subunit that translocates to activated RTKs upon interaction of its SH2 domains with pYXXM motifs, including Y_721_VEM in the CSF-1R [69, 84]. Upon binding, p85 activates p110 to produce phosphatidylinositol 3, 4, 5-trisphosphate (PIP_3_) from PI 4, 5-bisphosphate (PIP_2_) at the cell membrane [85]. An accumulation of PIP_3_ at the leading edge stimulates migration by inducing plasma membrane translocation of pleckstrin homology (PH) domain-containing molecules, PDK1, Akt, and Rho family GTPase regulators, or molecules with other PIP_3_ binding motifs such as WASP and its homologues [86, 87]. RTK-induced PIP_3_ levels are rapidly returned to baseline levels by the phospholipid phosphatase, PTEN [84]. While p110*α* and *β* are ubiquitously expressed, p110*δ* expression is highly enriched in hematopoietic cells, including macrophages. The three PI3K p110 isoforms have nonredundant biological roles and their function differs between primary and immortalised macrophages such that while p110*δ* is the main isoform recruited to the CSF-1R in bone marrow-derived macrophages, all three are recruited to the receptor in BAC1.2F5 macrophages [84]. However, PI3K p110*δ* appears to be the main regulator of migration in both primary macrophages and BAC1.2F5 cells, in which it triggers actin polymerization, cytoskeletal remodeling, and cell adhesion [84]. The exact pathways by which induction of PIP_3_ mediates these disparate effects of CSF-1 stimulation have yet to be identified, but regulation of individual elements further downstream in macrophage migration are becoming clearer and appear to converge on the Rho family GTPases.

## 6. Rho Family GTPases in Macrophage Motility

**Figure 3:**
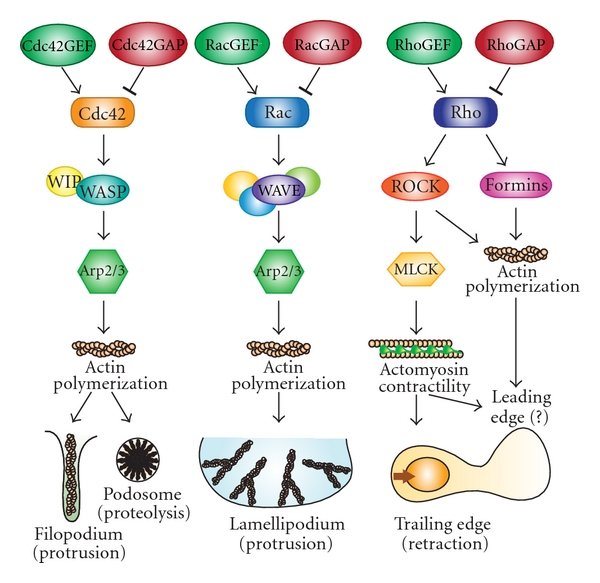
A schematic of signaling pathways activated by the Rho GTPases in macrophages. Generic GEFs and GAPs are designated as upstream regulators of Cdc42, Rac, and Rho activity. Downstream signaling molecules specifically labeled in the schematic are those that are described in the text. Signaling pathway outcomes are depicted for actin cytoskeleton responses only. Regulation of adhesion by Rho GTPases in macrophages is not shown as the signaling pathways are not yet elucidated.

Rho family GTPases are well-known regulators of actin polymerization and cell adhesion downstream of RTKs in many different cell types [88]. The main Rho family proteins found in macrophages are RhoA, RhoB, Rac1, Rac2, and Cdc42 [70]. Rho GTPases are activated by guanine-nucleotide exchange factors (GEFs), which stimulate the exchange of GDP for GTP, and inactivated by GTPase activating proteins (GAPs), which stimulate GTP hydrolysis (Figure 3). Upon activation, Rho family GTPases interact with effector proteins, including actin polymerization activators and protein kinases. Selective expression of GEFs, GAPs, and effector proteins plus spatiotemporal regulation of activation and regulatory crosstalk between Rho family proteins results in highly complex and dynamic coordination of cytoskeletal remodeling in response to RTK stimulation [88, 89]. Fluorescence resonance energy transfer (FRET) biosensors have been used to demonstrate in real time that the three ubiquitously expressed Rho family proteins, RhoA, Rac1, and Cdc42, are all activated at the leading edge of cells with very small differences in time and space [90, 91]. Rho family biosensors have yet to be used in CSF-1-dependent macrophage cell lines, which are difficult to transfect, but recent use of a WASP biosensor in RAW264.7/L5 macrophages demonstrated Cdc42-dependent activation of WASP in CSF-1-induced protrusions [92]. Early research into the role of Rho family proteins in macrophages used microinjection of constitutively active or dominant negative RhoA, Rac1, or Cdc42 to show that Cdc42 promoted filopodia formation while Rac stimulated ruffling and lamellipodial spreading and Rho triggered actomyosin contractility and retraction of the trailing edge in response to CSF-1 [93]. In addition, Rac and Cdc42 stimulated focal complex formation [94]. A subsequent study indicated that Rac and Rho were important for macrophage migration while Cdc42 regulated polarization and chemotactic sensing [94]. However, dominant negative proteins, particularly when overexpressed, may not be specific for their GEFs, and conditional knock-out and knock-down approaches have been used more recently to examine the role of Rho family proteins in macrophage actin remodeling and motility [70]. Although little is known about the loss of Cdc42 function, the effects of deletion of Rac1, which is ubiquitously expressed, and the hematopoietically restricted Rac2 have been reported [95–97]. Surprisingly, loss of both Rac1 and Rac2 did not decrease CSF-1-induced 2D motility, although Rac1/2^−/−^ macrophages did not form ruffles, normal lamellipodia, or podosomes [97]. Moreover, loss of Rac1 reduced invasive capacity of macrophages in Matrigel while loss of Rac2 reduced peritoneal macrophage infiltration in response to an inflammatory stimulus, suggesting both Rac proteins may be important for macrophage interstitial migration *in vivo* [96, 97]. In contrast to the unexpectedly mild loss-of-function phenotype of Rac1/2, C3 transferase-induced inhibition of RhoA-C inhibited CSF-1-stimulated macrophage migration and actomyosin contractility [40, 94]. Interestingly, global activation of Rac, Rho, or Cdc42 is not detected in CSF-1 stimulated macrophages at the time of the first wave of actin polymerization, suggesting local changes may be subtle [69]. Thus, the complexities and redundancies of Rho family GTPase signaling makes it difficult to tease apart the finer aspects of their role in CSF-1-induced motility. Production and examination of Cdc42^−/−^ and RhoA^−/−^ macrophages as well as FRET studies should prove illuminating. Nevertheless, further mechanistic insights into Rho family GTPase regulation of macrophage motility have been gained through examination of their main downstream effectors for actin polymerization, the WASP and WAVE complexes [71, 98].

In macrophages, Cdc42 and Rac stimulate actin polymerization by activating the Arp2/3 complex through their GTP-dependent association with the Arp2/3 activating scaffold proteins, WASP and WAVE, respectively [98, 99]. WASP was originally discovered as the hematopoietically expressed product of the gene mutated in the X-linked immunological disorder Wiskott Aldrich Syndrome (WAS) and is an important downstream effector of Cdc42 [92, 98, 99]. WAS myeloid cells display marked cytoskeletal abnormalities and cannot form podosomes, and WAS macrophages fail to chemotax towards CSF-1 [53, 73, 99]. WASP does not act in isolation and requires its N-terminal binding protein, WASP interacting protein (WIP), to form a functional unit that activates Arp2/3 in podosome formation and transendothelial migration in macrophages [99, 100]. Consistent with the requirement for both proteins to activate Arp2/3, WIP^−/−^ dendritic cells also fail to form podosomes, instead forming longer lived focal contacts [101]. The WAVE family proteins, WAVE1, 2, and 3, each stably complex with several other proteins in order to mediate the effects of Rac [71, 98]. WAVE2, which is the major WAVE isoform expressed in macrophages, is important for CSF-1 stimulation of ruffling and migration [74]. Thus, both WASP and WAVE2 activate the Arp2/3 complex to stimulate dendritic or branched actin polymerization, but WASP mediates regulation of chemotaxis by Cdc42, while WAVE2 mediates the regulation of ruffling and motility by Rac. Rather than activating Arp2/3, Rho promotes actomyosin contractility through activation of Rho-kinase(ROCK)1 and ROCK2. Unexpectedly however, ROCK1^−/−^ macrophages were more motile towards CSF-1 *in vitro *and responded to an inflammatory stimulus more readily *in vivo *[102]. Underlying their increased migration, ROCK1^−/−^ macrophages demonstrated increased adhesion on fibronectin and increased CSF-1-stimulated F-actin levels in association with increased PIP_3_ levels. ROCK1 was shown to negatively regulate CSF-1-induced migration through regulation of PTEN activity [102]. Rho also mediates its effects through activation of the formin family of actin nucleators, which assemble linear rather than branched actin filaments, but their role in macrophage motility is currently unknown [103].

The specific roles of individual Rho family proteins in the regulation of macrophage adhesion is less well understood than their roles in actin polymerization and actomyosin contractility and is made more complex by the fact that there is crosstalk between actin polymerization, actomyosin contractility, and adhesion formation and turnover [37, 104]. Nevertheless, CSF-1 stimulates the incorporation of the adhesion kinases, FAK and Pyk2, and their substrate, paxillin, into focal complexes and point contacts a few minutes after it stimulates actin polymerization, ruffling, and spreading in macrophages [40, 48, 69]. FAK is known to regulate adhesion formation and disassembly and both FAK and Pyk2 regulate macrophage migration *in vitro *and *in vivo*, apparently via the same pathway, as loss of Pyk2 does not further reduce migration and invasion in FAK^−/−^ macrophages [48, 72]. The precise mechanism by which CSF-1R signaling activates Pyk2 and FAK to regulate adhesion formation and turnover is not understood but CSF-1-stimulated FAK^−/−^ macrophages demonstrated high levels of Rac activity in association with hyperprotrusiveness [48] while Pyk2^−/−^ macrophages showed reduced integrin-mediated Rho activation [72]. These suggestions that adhesion signaling feeds back on actin polymerization and actomyosin contractility are not unexpected as many Rho family GEFs and GAPs are recruited to adhesions where they can activate or inhibit Rho family proteins [41]. Moreover, activated and autophosphorylated FAK and Pyk2 associate with SFKs in adhesions, facilitating SFK-based phosphorylation and regulation of nearby Rho family GEFs and GAPs as well as other adhesion proteins [104]. Phosphorylation of paxillin triggers its translocation to adhesions and brings along associated Rac effectors that are critical for leading edge formation and adhesion turnover [43, 47]. Thus, adhesions themselves are important platforms for the regulation of Rho family proteins [37, 104].

An area where the role of individual Rho GTPases has been more clearly defined in macrophages is phagocytosis, which is a highly ordered process of membrane protrusion and actin polymerization that uses many of the same elements of cellular machinery as locomotion [105]. FRET studies of Fc*γ* receptor-mediated phagocytosis in RAW264.7 macrophages reveal distinct spatiotemporally regulated patterns of Rac1, Rac2, and Cdc42 activation underlying actin polymerization in the phagocytic cup [106]. In addition, RhoG, which is more closely related to Rac than to Rho [107], is recruited to phagocytic cups in J774 macrophages [108]. Rho GTPases activate many of the same downstream effectors in phagocytosis as they do in motility, with both WASP and Arp2/3 being required for normal Fc*γ* receptor-mediated phagocytosis in macrophages [105]. Further refinements in the application of FRET to CSF-1-dependent mature macrophages will reveal the specific spatiotemporally regulated roles of individual Rho GTPases in adhesion, motility, and phagocytosis.

## 7. Concluding Remarks

It is clear that the interplay between the different elements of the adhesion and motility apparatus, coordinated in large part by Rho family GTPases, is complex and will require the use of many different approaches to unpack these complexities. Mature macrophages have proven difficult to adapt to some approaches as, not only do they selectively express a number of important adhesion and motility proteins but they are difficult to transfect [1]. Nevertheless, motile macrophages contribute to the progression of a number of important diseases and elucidation of how CSF-1 regulates polarization, protrusion, adhesion, actomyosin contractility, and trailing edge retraction to stimulate migration is important in the development of therapies to treat these diseases. The CSF-1R-deficient mouse macrophage cell line (MacCsf1r^−/−^), when transduced with individual wild-type or tyrosine mutant CSF-1Rs, allows examination of specific signaling pathways triggered by individual tyrosine residues in mature macrophages [77]. Using this system, Y721 was recently identified as the major CSF-1R phosphotyrosine residue triggering ruffling, adhesion, and motility in response to CSF-1 [69]. Furthermore, the primary mediator of pY721-based signaling to motility was demonstrated to be PI3K [69], and work is now focused in identifying the specific PI3K p110 isoform and PI3K-activated pathways that regulate actin polymerization, adhesion formation, and migration in macrophages. CSF-1R and isoform specific PI3K inhibitors are available and may prove useful in the treatment of disseminated tumors and chronic inflammatory arthritides.

## Abbreviations

CSF-1: Colony-stimulating factor-1
CSF-1R: Colony-stimulating factor-1 receptor
EGF: Epidermal growth factor
EM: Electron microscopy
FAK: Focal adhesion kinase
FRET: Fluorescence resonance energy transfer
GEF: Guanine-nucleotide exchange factor
GAP: GTPase activating protein
IL-34: interleukin-34
MLCK: Myosin light chain kinase
op: Osteopetrotic
PDGFR: Platelet derived growth factor receptor
PH: Pleckstrin homology
PI3K: Phosphoinositol 3′-kinase
PIP3: Phosphatidylinositol 3, 4, 5, trisphosphate
PLC*γ*: Phospholipase C*γ*
pTyr: Phosphotyrosine
ROCK: Rho kinase
RTK: Receptor tyrosine kinase
SFK: Src family kinase
TIRF: Total internal reflection fluorescence microscopy
WASP: Wiskott Aldrich Syndrome protein
WAVE: WASP-family verprolin homologous
WIP: WASP-interacting protein.
